# ^177^Lu-Lilotomab Satetraxetan Has the Potential to Counteract Resistance to Rituximab in Non-Hodgkin Lymphoma

**DOI:** 10.2967/jnumed.119.237230

**Published:** 2020-10

**Authors:** Marion M. Malenge, Sebastian Patzke, Anne H. Ree, Trond Stokke, Peter Ceuppens, Brian Middleton, Jostein Dahle, Ada H.V. Repetto-Llamazares

**Affiliations:** 1 Nordic Nanovector ASA, Oslo, Norway; 2 Department of Radiation Biology, Institute for Cancer Research, Oslo University Hospital, Oslo, Norway; 3 Institute of Clinical Medicine, University of Oslo, Oslo, Norway; 4 Department of Oncology, Akershus University Hospital, Lørenskog, Norway; and; 5 Inferstats Consulting Ltd., Cheshire, United Kingdom

**Keywords:** ^177^Lu, radioimmunotherapy, NHL, rituximab resistance

## Abstract

Patients with non-Hodgkin lymphoma (NHL) who are treated with rituximab may develop resistant disease, often associated with changes in expression of CD20. The next-generation β-particle–emitting radioimmunoconjugate ^177^Lu-lilotomab-satetraxetan (Betalutin) was shown to up-regulate CD20 expression in different rituximab-sensitive NHL cell lines and to act synergistically with rituximab in a rituximab-sensitive NHL animal model. We hypothesized that ^177^Lu-lilotomab-satetraxetan may be used to reverse rituximab resistance in NHL. **Methods:** The rituximab-resistant Raji2R and the parental Raji cell lines were used. CD20 expression was measured by flow cytometry. Antibody-dependent cellular cytotoxicity (ADCC) was measured by a bioluminescence reporter assay. The efficacies of combined treatments with ^177^Lu-lilotomab-satetraxetan (150 or 350 MBq/kg) and rituximab (4 × 10 mg/kg) were compared with those of single agents or phosphate-buffered saline in a Raji2R-xenograft model. Cox regression and the Bliss independence model were used to assess synergism. **Results:** Rituximab binding in Raji2R cells was 36% ± 5% of that in the rituximab-sensitive Raji cells. ^177^Lu-lilotomab-satetraxetan treatment of Raji2R cells increased the binding to 53% ± 3% of the parental cell line. Rituximab ADCC induction in Raji2R cells was 20% ± 2% of that induced in Raji cells, whereas treatment with ^177^Lu-lilotomab-satetraxetan increased the ADCC induction to 30% ± 3% of that in Raji cells, representing a 50% increase (*P* < 0.05). The combination of rituximab with 350 MBq/kg ^177^Lu-lilotomab-satetraxetan synergistically suppressed Raji2R tumor growth in athymic Foxn1^nu^ mice. **Conclusion:** ^177^Lu-lilotomab-satetraxetan has the potential to reverse rituximab resistance; it can increase rituximab binding and ADCC activity *in vitro* and can synergistically improve antitumor efficacy *in vivo*.

Non-Hodgkin lymphoma (NHL) is the most common hematologic malignancy and had the 11th highest mortality rate of all malignancies worldwide in 2018 (1,2). B lymphocytes are predominantly the origin of NHL, with malignant B cells expressing a high density of specific antigens such as CD20 and CD37 on their surface (3). These antigens provide a platform for antibody-based targeted therapies (4). Immunotherapy with the CD20-directed antibody rituximab inhibits cell proliferation by inducing antibody-dependent cellular cytotoxicity (ADCC) and complement-dependent cytotoxicity (5). Although rituximab alone and in combination with chemotherapy are a mainstay of NHL treatment (6–8), the efficacy is variable (9). Some patients are reported to have disease progression after an initial response to rituximab (10). Conversely, rituximab-naïve patients have been reported with primarily rituximab-refractory disease (11).

The mechanisms of rituximab resistance are not completely understood (9,12). Rituximab resistance is postulated to be a result of downregulation of the *CD20* gene, internalization, lysosomal degradation, and shaving off of rituximab/CD20 complexes (13–18).

Strategies to counteract rituximab resistance include combination therapies and targeting of alternative antigens. Previous studies have described the ability of ionizing radiation to potentiate immunotherapy through the generation of reactive-oxygen species that mediate an increase in antigen expression (19–21), consequently improving on antibody-dependent toxicity in addition to the direct cytotoxic radiation effect (21,22). Anti-CD20 antibody binding increased up to 2-fold, 20–120 h after irradiation (19,20,23). Radioimmunotherapy delivers targeted short-range radiation that effectively ablates malignant cells and with limited toxicity to normal tissues (24,25).

The anti-CD37 radioimmunoconjugate ^177^Lu-lilotomab-satetraxetan (^177^Lu-lilotomab), consisting of the β-emitting isotope ^177^Lu (half-life, 6.7 d) chelated to the chemical linker p-SCN-benzyl-DOTA (satetraxetan) conjugated to the murine antibody lilotomab, has shown robust antitumor activity and low toxicity in preclinical models (26,27). ^177^Lu-lilotomab is currently in clinical trials for relapsed or refractory lymphomas (NCT01796171 and NCT02658968) (25,28).

We have recently shown that pretreatment of rituximab-sensitive NHL cells with ^177^Lu-lilotomab increases CD20 binding *in vitro* and synergistically increases the antitumor effect when combined with rituximab *in vivo* (23). Currently, ^177^Lu-lilotomab is being tested in combination with rituximab in patients with previously treated follicular lymphoma (NCT03806179).

Here, we hypothesized that ^177^Lu-lilotomab can reverse rituximab resistance in NHL. We used a rituximab-resistant NHL cell line and animal model and explored the mechanism of synergy by measuring rituximab binding, ADCC induction and apoptosis.

## MATERIALS AND METHODS

### Cell Lines

The Burkitt lymphoma cell lines Raji and Raji2R, from Roswell Park Institute (16), were cultured in RPMI medium (ThermoFisher) supplemented with GlutaMAX, 10% heat-inactivated fetal bovine serum, and 1% penicillin–streptomycin at 37°C with 5% CO_2_.

### Radiolabeling of Antibodies with ^177^Lu

Lilotomab-satetraxetan was pH-adjusted using ammonium acetate and then radiolabeled with ^177^Lu (ITG) at 37°C for 15–30 min. The specific activity for all *in vitro* studies was 600 MBq/mg, whereas 200 MBq/mg was chosen for *in vivo* studies. The radiochemical purity and immunoreactive fraction of the conjugate were determined using instant thin-layer chromatography and a method modified from that of Lindmo et al. (29), respectively.

### Measurement of CD20 Binding

Cells at a concentration of 2.5 × 10^6^/mL were incubated for 18 h with 0–20 μg/mL of lilotomab, ^177^Lu-lilotomab, or phosphate-buffered saline (PBS, control) at 37°C. The cells were then washed, resuspended in fresh medium to a concentration of 0.5 × 10^6^/mL and cultured for up to 6 days, with fresh medium added on day 3. On days 3 and 6, the cells were prepared for flow cytometric assays using rituximab (Roche) conjugated to Alexa-Fluor647 tetra fluorophenyl ester (ThermoFisher) according to the manufacturer’s instructions. The cell concentration was adjusted to 1 × 10^6^/mL, and Raji cells were stained with 0.4 μg/mL Hoechst 33342 (Life Technologies) for identity barcoding at 37°C for 20 min and then washed using ice-cold PBS. To assess the effect of ^177^Lu-lilotomab treatment on CD20 binding, the cells were incubated at 4°C with 30 μg/mL rituximab-Alexa647 for 30 min. To estimate the background signal, the cells were incubated with a 100-fold excess of nonfluorescent rituximab before addition of rituximab-Alexa647.

The cells were washed, and fluorescence was read by flow cytometry (Guava easyCyte12HT; Millipore). Changes in rituximab binding on ^177^Lu-lilotomab–treated cells relative to control cells for each cell line were assessed using Equation 1.

Eq. 1 $$\text{Increase in rituximab binding}=\frac{\text{rituximab binding}\;\left(\text{treated}-\text{control}\right)\;\text{cells}\;\;}{\text{rituximab binding }\left(\text{control}\right)\;\text{cells}}\times 100$$

Rituximab binding in rituximab-resistant Raji2R cells (control and treated cells) was compared with rituximab binding in untreated (control) rituximab-sensitive Raji cells using Equation 2.

Eq. 2 $$\text{Relative rituximab binding}=\frac{\text{rituximab binding of Raji}2\text{R}}{\text{rituximab binding of Raji }\left(\text{control}\right)}\times 100$$

### Measurement of ADCC

The cells at a concentration of 2.5 × 10^6^/mL were incubated with 1 μg/mL of lilotomab, ^177^Lu-lilotomab, or controls at 37°C for 18 h. All cells were washed and adjusted to 0.5 × 10^6^/mL in fresh medium before being further incubated. After 6 days, rituximab-induced ADCC activity was measured using ADCC reporter bioassay kits (Promega) containing Jurkat cells engineered to stably express FcγRIIIa receptor (30) as effector cells. These cells have a firefly luciferase gene driven by a nuclear-factor-of-activated-T-cell response element reporting the activation of the gene by producing luciferase quantified as luminescence signal. The cells were coincubated with 0.68–40 μg/mL rituximab and effector cells for 22 h at a 2:1 effector-to-target cell ratio. ADCC activity was measured as the luminescence of cell-bound effector cells. The change in ADCC induction by rituximab in ^177^Lu-lilotomab–treated cells relative to control cells was obtained using Equation 3.

Eq. 3 $$\text{Increase in ADCC induction}=\frac{\text{ADCC induction in cells}\;\left(\text{treated}-\text{control}\right)\;}{\text{ ADCC induction in cells }\left(\text{control}\right)}\times 100$$

Relative ADCC induction by rituximab in Raji2R control and ^177^Lu-lilotomab–treated cells to Raji control cells was obtained using Equation 4.

Eq. 4 $$\text{Relative ADCC induction}=\frac{\text{ADCC induction in Raji}2\text{R}}{\;\text{ADCC induction in Raji}\;\left(\text{control}\right)}\times 100$$

### Measurement of Cell Viability and Apoptosis

At a concentration of 2.5×10^5^/mL, Raji and Raji2R cells were incubated at 37°C with either 50 µg/mL rituximab or PBS. At 1 h and 3 days after the start of incubation, the cells were transferred to 96-well plates and incubated with the RealTime-Glo MT Cell Viability Assay (Promega) following the manufacturer’s protocol. The luminescence, proportional to the number of viable cells, was measured at each time point on a Spark microplate reader (Tecan). The experiment was performed in duplicates, and the results are presented as mean ± SD.

On day 3, 2.0 × 10^6^ cells were fixed using ice-cold methanol in preparation for evaluation of apoptosis by flow cytometry analysis. A positive control was included in the study by incubating the unfixed control cells with a topoisomerase inhibitor, etoposide, for 18 h before analysis. The fixed cells were then washed and incubated with Alexa-conjugated anticleaved Poly (ADP-ribose) polymerase (PARP) antibody (BioNordika) diluted 1:100 in 5% nonfat milk for 1 h. The cells were once again washed, and the fluorescent apoptosis signal was determined by flow cytometry (Guava easyCyte12HT; Millipore).

### *In Vivo* Xenograft Model

All procedures in this study were approved by the Norwegian Animal Research Authority and performed in accordance with Norwegian Animal Research Authority regulations and Federation of European Laboratory Animal Science Associations recommendations.

Female athymic nude Foxn1^nu^ mice bred at the Institute for Comparative Medicine, Oslo University Hospital, Norway, were used.

The mice, aged 4–5 wk old with an average weight of 21 ± 2 g, were injected subcutaneously in both flanks with 10 × 10^6^ Raji2R cells per flank using a 1:1 Matrigel (Corning) dilution ratio. The mice were injected intraperitoneally with 50 μL of anti-asialo GM1 (Wako Chemicals) after dilution per the manufacturer’s recommendation, 24 h before cell inoculation and once every week thereafter for the rest of the study. This was administered to increase tumor take and prevent spontaneous tumor regression by decreasing the Natural Killer (NK) cell population in the mice. On attaining a tumor diameter between 4 and 11 mm, the mice were placed into treatment groups of 10 mice each, ensuring similar average tumor volumes per group.

### Therapy Study

Raji2R-xenografted mice were administered NaCl, rituximab monotherapy as 4 subsequent doses every 3–4 d (4 × 10 mg/kg), 150 MBq/kg ^177^Lu-lilotomab as monotherapy, 350 MBq/kg ^177^Lu-lilotomab as monotherapy, combination therapy with 150 MBq/kg ^177^Lu-lilotomab and rituximab (4 × 10 mg/kg), and combination therapy with 350 MBq/kg ^177^Lu-lilotomab and rituximab (4 × 10 mg/kg). The dosing concentrations of ^177^Lu-lilotomab were below the maximum tolerated dose (∼550 MBq/kg) in nude mice (27). The two chosen dosing concentrations were considered to be therapeutically suboptimal without the combination with rituximab, which would make it feasible to observe any synergistic effect of the combination.

Caliper measurements of the tumors in three dimensions were recorded 2–3 times a week. Tumor volume was calculated as $\frac{\text{π}}{6}\left(\text{length × width × height}\right)$. Animal health status was monitored for the length of the study, and the animals were euthanized by cervical dislocation when tumor diameter was more than 20 mm or if they were observed to experience severe poor health, tumor necrosis or ulceration, weight gain, or more than a 10% loss from the maximum or minimum recorded weight or any other signs of discomfort. After euthanasia, the animal was dissected to check for any anatomic anomalies.

### Statistical Analysis

*In vitro* data were analyzed in SigmaPlot (version 13.0; Systat) and Prism (version 8; GraphPad) using 2-tailed *t* tests on either complete datasets or paired averaged data, to compare the different groups, cell lines, and time points. Data are presented as mean ± SE, and a *P* value of less than 0.05 was considered statistically significant.

Mouse survival was defined as the time until death due to a tumor diameter of more than 20 mm (representative of disease progression). An alternative analysis was performed by defining mice survival as the time until death due to either a tumor diameter of more than 20 mm or tumor ulceration. The analysis was performed in SigmaPlot using the log-rank test, reporting statistical significance by the Holm–Sidak test for multiple comparisons.

Tumor volume was computed in two different ways: as the average ± SE for each group, maintaining tumor volume constant after euthanasia along the 70 d of the study, and by extrapolation of tumor volume after euthanasia, which is considered a better representation of the data but can only be performed up to 20 days because tumor volumes become infeasibly large. SAS (version 9.4; SAS Institute Inc.) was used for these calculations.

### Bliss Independence Analysis of Mouse Survival

Bliss analysis of mouse survival was performed by fitting a Cox proportional-hazards model to the survival data. The Bliss definition of synergy was assessed by the interaction of the combination treated groups with the rituximab and respective ^177^Lu-lilotomab monotherapy groups. Interaction values lower than 1 were considered synergistic, and statistical significance was defined both by a *P* value of less than 0.05 and equivalently by the upper limit of the 90% confidence interval being less than 1. R (2019) with survival package was used for these calculations.

### Bliss Independence Analysis of Tumor Volume

Bliss analysis of tumor volume was performed using extrapolation of tumor volumes and was restricted to the first 20 days of the study because there were no control animals beyond study day 13 and any analysis beyond day 20 would impose uncertainty. The tumor volumes were log-transformed, and data for mice withdrawn before study day 20 were extrapolated by linear regression. Beyond day 20, tumor sizes become infeasibly large. The difference from baseline was calculated on the log‐scale, and all statistical analyses were performed on the log-transformed data. A mixed-effects linear model was used, including fixed effects of each of the treatments (referred to as between-group factors) and the associated interaction between these factors. Additionally, study day was included as a within-animal fixed effect. All the interactions between the group factors and study day were included. Animal ID and the side of the tumor (right or left) were included as random effects in the model. An autoregressive correlation structure was assumed. The effects of treatment with and without rituximab were evaluated separately at each dose of ^177^Lu-lilotomab (control, 150 MBq/kg, and 350 MBq/kg) for each study day. The size of these effects was compared for 150 MBq/kg ^177^Lu-lilotomab against the control and for 350 MBq/kg ^177^Lu-lilotomab against the control using the interaction test of the Bliss independence model using SAS, version 9.4. Interaction values of less than 1 were considered synergistic, and statistical significance was defined by a *P* value of less than 0.05.

## RESULTS

### Increased Rituximab Binding by ^177^Lu-Lilotomab

**FIGURE 1.**
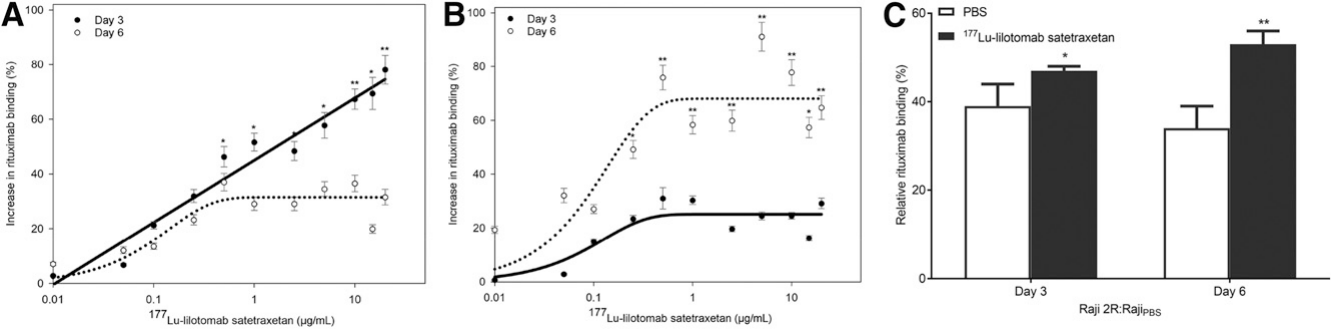
(A and B) Increase in rituximab binding on days 3 and 6 after treatment with escalating doses of ^177^Lu-lilotomab in Raji cells (A) and Raji2R cells (B). (C) Rituximab binding in Raji2R cells relative to untreated Raji cells when considering average of horizontal plateau from B. *P < 0.05. **P < 0.005. *n* = 3.

Exposure of Raji and Raji2R cells to ^177^Lu-lilotomab resulted in a dose-dependent increase in rituximab binding as compared with control cells (Fig. 1). The increase in rituximab binding (Eq. 1) was fitted using a regression line based on the 2-parameter-exponential-rise-to-maximum equation (*R*^2^ values between 0.71 and 0.90). Rituximab binding in ^177^Lu-lilotomab–treated Raji cells continuously increased when compared with the control, reaching 78% 3 days after treatment (Fig. 1A). Six days after treatment, rituximab binding showed an initial exponential increase from the control, followed by a plateau at 31% for ^177^Lu-lilotomab concentrations above 0.5 μg/mL. The same was observed in Raji2R cells, with a plateau at 25% for 3 days and at 68% for 6 days after ^177^Lu-lilotomab treatment (Fig. 1B). The increase in rituximab binding at 3 days was significantly different from that at 6 days in both cell lines (*P* < 0.01). Binding in Raji cells was highest at 3 days after treatment, whereas in Raji2R cells it was highest at 6 days.

To compare the relative rituximab binding of Raji2R versus Raji cells (Eq. 2), the maximum asymptote of the fitted curves in Figure 1B was used. Rituximab binding in Raji2R cells was on average 36% ± 5% of the binding in Raji cells when no ^177^Lu-lilotomab was given (Eq. 2; Fig. 1C).

After treatment with ^177^Lu-lilotomab, the relative binding to Raji2R cells compared with untreated Raji cells increased to 47% ± 1% (*P* < 0.01) at 3 days and 53% ± 3% (*P* < 0.01) at 6 days. In contrast, treatment with unlabeled lilotomab or PBS had no effect on rituximab binding.

### Enhanced ADCC by Rituximab After ^177^Lu-Lilotomab Treatment

**FIGURE 2.**
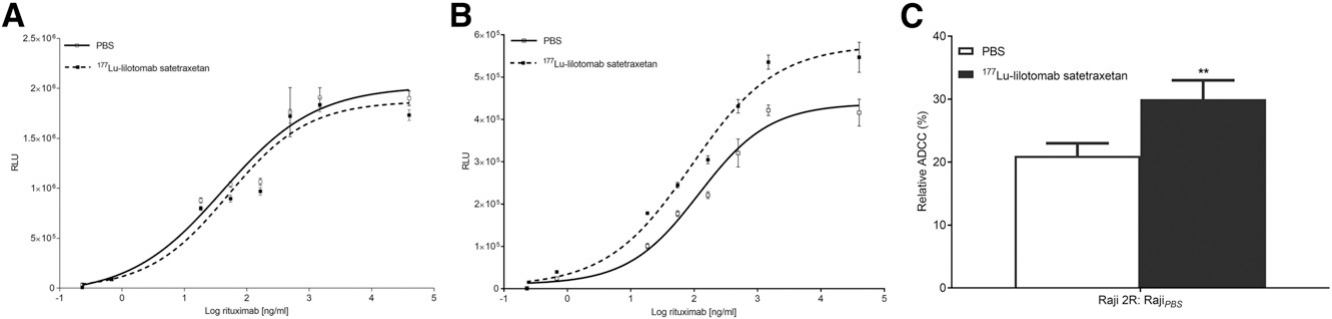
(A and B) Luminescence (RLU) representative of effector-cell binding to rituximab in Raji cells (A) and Raji2R cells (B) treated with 1 μg/mL ^177^Lu-lilotomab or PBS (untreated). (C) Relative change in effector-cell binding to rituximab in untreated and in 1 μg/mL ^177^Lu-lilotomab–treated Raji2R cells relative to untreated Raji cells. **P < 0.05. *n* = 3–4.

ADCC induction was assessed by measurement of effector-cell binding of cell-bound rituximab in cells previously treated with ^177^Lu-lilotomab or PBS (control). There was no significant change in effector-cell binding of rituximab in Raji cells after treatment with ^177^Lu-lilotomab (Fig. 2A, *P* > 0.05). Conversely, treatment of Raji2R cells with ^177^Lu-lilotomab significantly augmented effector-cell binding (*P* < 0.05, Fig. 2B). The maximum asymptote of the fitted curves from Figures 2A and B was used to calculate the increase in ADCC induction and the relative increase in ADCC induction in Raji2R versus Raji cells (Eqs. 3 and 4, respectively). Effector-cell binding increased by 47% ± 4% in ^177^Lu-lilotomab–treated Raji2R cells compared with untreated Raji2R cells (Eq. 3). Effector-cell binding in ^177^Lu-lilotomab–treated Raji2R cells was 43% higher than that in untreated Raji2R cells relative to untreated Raji cells (30% ± 3% vs. 21% ± 2%, *P* < 0.05; Eq. 4 and Fig. 2C). Unlabeled lilotomab did not modulate effector-cell binding.

### Effect of Rituximab Treatment on Cell Viability and Apoptosis

Treatment with rituximab did not yield any significant effect on cell viability relative to the untreated cells after 1 h and on day 3 for either the Raji and the Raji2R cells (Supplemental Fig. 1; supplemental materials are available at http://jnm.snmjournals.org). In addition, treatment of Raji and Raji2R cells with rituximab had no significant effect on initiating apoptosis. The percentage of total number of apoptotic cells in rituximab-treated Raji cells was similar to that in the untreated control cells at day 3 (Supplemental Fig. 2). Raji 2R cells were overall resistant to rituximab treatment, and no apoptosis was observed (Supplemental Fig. 2).

### Synergistic Antitumor Efficacy of Combination of ^177^Lu-Lilotomab and Rituximab

#### Analysis Based on Tumor Growth

**FIGURE 3.**
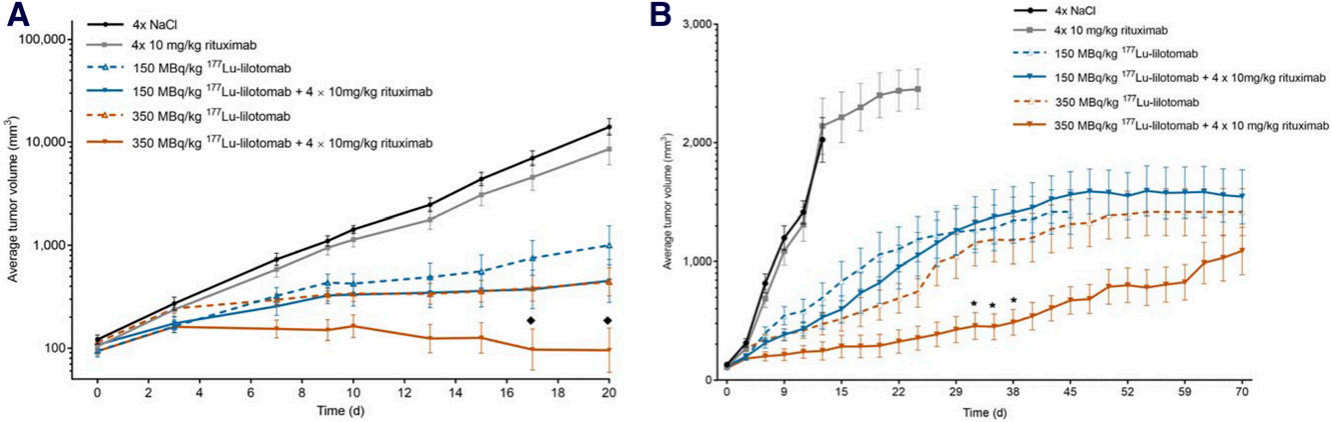
Average tumor volume ± SE in Raji2R-xenografted mice treated with PBS, rituximab, and 150 and 350 MBq/kg concentrations of ^177^Lu-lilotomab as monotherapy or in combination with rituximab. *n* = 10. (A) Curve built using extrapolation of tumor volumes after euthanasia. ♦ = timepoints of observed significant synergistic effects (*P* < 0.05). (B) Curve built keeping constant tumor volume after euthanasia. * = timepoints observed to be significantly different from ^177^Lu-lilotomab monotherapy (*P* < 0.05).

Treatment of Raji2R-xenografted mice with rituximab alone did not suppress tumor growth compared with that in mice treated with PBS (Fig. 3). However, treatment with ^177^Lu-lilotomab alone or in combination with rituximab showed inhibition of tumor growth when compared with the PBS and rituximab-treated tumors. This inhibition was reflected in the lower fold change in tumor volume from baseline at various time points after the start of treatment with the combination of 150 or 350 MBq/kg ^177^Lu-lilotomab and rituximab, compared with monotherapy with the respective treatments (*P* < 0.05; Table 1). The Bliss independence model indicated significant synergism in combining 350 MBq/kg ^177^Lu-lilotomab with rituximab (*P* < 0.05 for tumor volumes measured 17 and 20 days after treatment), whereas the combination of 150 MBq/kg ^177^Lu-lilotomab and rituximab did not reach statistical significance for any time point (Fig. 3A). When analyzing the tumor volume data during the study (by maintaining the last tumor volume after euthanasia), we found a significant difference between the 350 MBq/kg ^177^Lu-lilotomab monotherapy and the respective combination with rituximab (*P* < 0.05; Fig. 3B), indicating that ^177^Lu-lilotomab potentiated the rituximab effect.

**TABLE 1:** Fold Change in Average Tumor Volume from Baseline of Combination Therapies vs. Corresponding Monotherapies and Bliss Synergy Interaction Values

	^177^Lu-lilotomab (MBq/kg)	Fold change from day 0		
Study day		Without rituximab	With rituximab	Interaction value
3	0	2.2	2.2	
	150	1.7	1.6	0.99 (0.41, 2.37)
	350	2.3	1.7	0.77 (0.32, 1.84)
7	0	6.1	5.5	
	150	3.3	2.4	0.80 (0.33, 1.91)
	350	2.7	1.6	0.66 (0.28, 1.57)
9	0	9.2	8.9	
	150	4.4	3.0	0.70 (0.29, 1.68)
	350	3.1	1.6^†^	0.53 (0.22, 1.23)
10	0	11.8	10.6	
	150	4.3	3.1	0.79 (0.33, 1.89)
	350	3.2	1.7	0.61 (0.26, 1.46)
13	0	20.4	16.6	
	150	5.0	3.2	0.79 (0.33, 1.89)
	350	3.1	1.3^†^	0.51 (0.22, 1.23)
15	0	36.2	28.8	
	150	5.7	3.3	0.74 (0.31, 1.77)
	350	3.3	1.3^†^	0.50 (0.21, 1.21)
17	0	57.8	42.6	
	150	7.7	3.5*	0.61 (0.25, 1.47)
	350	3.5	1.0^†^	0.39 (0.16, 0.94)^‡^
20	0	116.6	80.2	
	150	10.2	4.2*	0.60 (0.25, 1.43)
	350	4.1	1.0^†^	0.36 (0.15, 0.86)^‡^

* Significant rituximab effect with 150 MBq/kg ^177^Lu-lilotomab (*P* < 0.05).

† Significant rituximab effect with 350 MBq/kg ^177^Lu-lilotomab (*P* < 0.05).

‡ Significant synergism (*P* < 0.05).

Data in parentheses are 90% confidence intervals.

#### Survival Analysis with End Point: Death Due to Tumor Diameter Larger Than 20 mm

**FIGURE 4.**
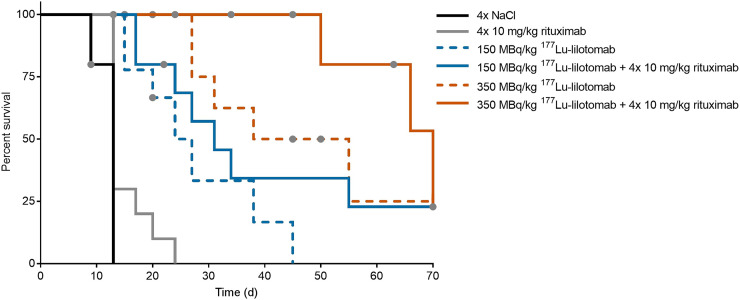
Kaplan–Meier survival curves of Raji2R-xenografted mice treated with PBS, rituximab, and 150 and 350 MBq/kg concentrations of ^177^Lu-lilotomab as monotherapy or in combination with rituximab. *n* = 10. Endpoint is tumor diameter larger than 20 mm. Gray dots = censored animals.

Treatment with ^177^Lu-lilotomab alone and in combination with rituximab significantly prolonged the time to event compared with PBS and rituximab treatment (Fig. 4; Table 2). The median survival time of mice treated with the combination of 350 MBq/kg ^177^Lu-lilotomab and rituximab was doubled when compared with survival in mice given 350 MBq/kg ^177^Lu-lilotomab monotherapy, and it was 5 times longer than for mice given rituximab monotherapy.

**TABLE 2:** Median Survival Time of Mice Treated with NaCl, Rituximab, 150 and 350 MBq/kg Concentrations of ^177^Lu-Lilotomab and Combination Therapies with 20-mm Tumor Diameter as Endpoint

Treatment group	Median survival ± SE (d)
4×NaCl	13 ± 0
4 × 10 mg/kg rituximab	13 ± 3
150 MBq/kg ^177^Lu-lilotomab	24 ± 4*^†^
350 MBq/kg ^177^Lu-lilotomab	38 ± 11*^†^
150 MBq/kg ^177^Lu-lilotomab + rituximab	31 ± 5*^†^
350 MBq/kg ^177^Lu-lilotomab + rituximab	70 ± 8*^†^

* Significantly different from NaCl (*P* < 0.001).

† Significantly different from 4 × 10 mg/kg rituximab (*P* < 0.01).

Bliss independence analysis did not provide statistically significant results (Table 3). The lack of significance might be due to the large number of censored animals and the poor proportional-hazards assumption in the Cox model (*P* = 0.048).

**TABLE 3:** Bliss Synergy Interaction Values Calculated Using Hazards Found Through Cox Proportional-Hazards Model Fitting to Mice Survival

Treatment group	Interaction value	*P*
150 MBq/kg ^177^Lu-lilotomab + rituximab	0.88 (0.30–2.63)	0.85
350 MBq/kg ^177^Lu-lilotomab + rituximab	0.83 (0.22–3.15)	0.82

Endpoint is tumor diameter larger than 20 mm. Data in parentheses are 90% confidence intervals.

**FIGURE 5.**
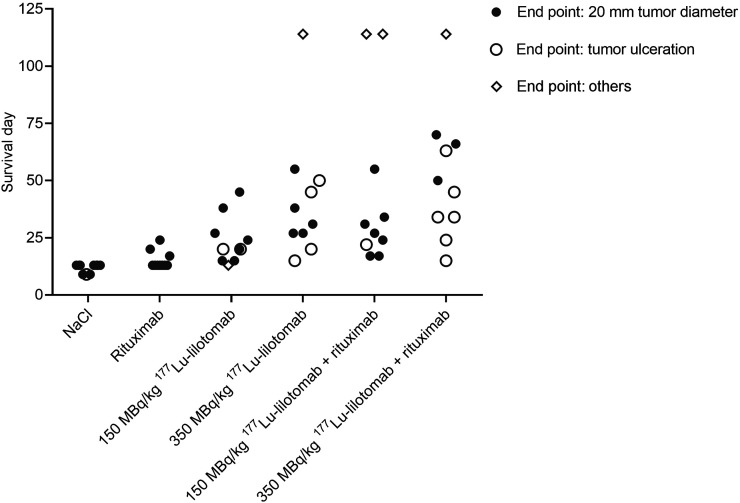
Survival of Raji2R-xenografted mice treated with PBS, rituximab, and 150 and 350 MBq/kg concentrations of ^177^Lu-lilotomab as monotherapy or in combination with rituximab. Diamonds represent mice euthanized because study ended (at 114 d) or because there were symptoms of sickness or discomfort.

In total, 14 of the 60 mice included in the study were euthanized because of tumor ulceration (Fig. 5). Most of the ulcers appeared in mice given ^177^Lu-lilotomab monotherapy or the combination with rituximab. These mice were regarded as censored in the survival analysis since the tumors did not reach the primary endpoint (tumor diameter > 20 mm).

#### Survival Analysis with End Point: Death Due to Tumor Diameter Larger Than 20 mm or Tumor Ulceration

Treatment with ^177^Lu-lilotomab alone and in combination with rituximab significantly prolonged survival compared with PBS and rituximab treatment (Supplemental Fig. 3 and Supplemental Table 1). However, treatment with ^177^Lu-lilotomab in combination with rituximab did not significantly differ from treatment with rituximab alone.

Bliss independence analysis did not provide statistically significant results (Supplemental Table 2). However, with only 10 mice per group, the hazard proportionality is an approximation. The lack of significance is because of the poor proportional-hazards assumption in the Cox model (*P* = 0.07).

## DISCUSSION

Although immunotherapy with rituximab has been widely successful, rituximab resistance in subsets of NHL patients remains a challenge in clinical management of the disease. In the present study, we demonstrated that *in vitro* treatment of rituximab-resistant Raji2R cells with ^177^Lu-lilotomab increased both rituximab binding and ADCC activity. In addition, we showed that *in vivo* combination of ^177^Lu-lilotomab with rituximab can synergistically suppress tumor growth in Raji2R-xenografted mice.

Evidence supports that ADCC activity may be the predominant *in vivo* mechanism of action of rituximab (31,32). We have therefore explored if ^177^Lu-lilotomab can restore ADCC by rituximab in the rituximab-resistant Raji2R cell line. Our findings show that partial restoration can be reached. The increased ADCC may be caused by the significant time-dependent increase in rituximab binding, an observation in line with results presented by Hiraga et al. (13), who hypothesized the delay to be due to altered transcriptional regulation resulting from persistent rituximab treatment during acquisition of resistance. In agreement with observations by van Meerten et al. (33), the direct cytotoxic or apoptotic effect of rituximab in the rituximab-sensitive Raji cells was negligible (Supplemental Figs. 1 and 2), and it was therefore not possible to study the sensitization of rituximab-resistant cells to rituximab by ^177^Lu-lilotomab using this model. Further studies using other rituximab-resistant cell lines are warranted.

Translation of the *in vitro* results to a clinical setting is limited. The dose delivered from ^177^Lu-lilotomab to cells in the *in vitro* studies is a function of both specific and nonspecific irradiation of the cells during the 18-h incubation time (34). Given that CD20 upregulation is mediated by intracellular redox regulation and is dose-dependent (19,23), we expect that treatment with a nonspecific radioimmunoconjugate will produce a similar increase in CD20 binding and a subsequent ADCC increase in this experimental set-up. However, in an *in vivo* or clinical setting, ^177^Lu-lilotomab would have an important advantage over a nonspecific radioimmunoconjugate due to its capability to deliver targeted radiation to tumor while sparing the healthy tissues.

Although the time to event for mice treated with the combination of ^177^Lu-lilotomab and rituximab was not significantly synergistic, there was significant synergy in tumor growth delay. We have shown that *in vivo* combination therapy with ^177^Lu-lilotomab and rituximab has the potential to synergistically suppress tumor growth in Raji2R-xenografted mice. The increased rituximab binding and enhanced ADCC shown .in our *in vitro* studies are among the mechanisms of action that could lead to the observed synergy.

Other mechanisms that might contribute to the observed synergy are improved complement-dependent cytotoxicity by colocalization of CD37 and CD20 on the cell membrane (35), radiation-induced permeability of tumor vasculature (36), radiation-induced immunogenic modulation of tumor cells (37–39), rituximab-induced sensitization of tumor cells to ionizing radiation (40), and rituximab-induced increased internalization of CD37 (41) leading to increased cellular retention of ^177^Lu and thus to a higher cellular absorbed radiation dose (25).

To have good tumor take and growth, our animal model required use of anti-asialo GM1 antibody to decrease the number of NK cells, which are the classic mediators of ADCC. This intentional decrease in NK cell numbers might have led to a reduced ADCC effect. The observed ADCC effect in our animal studies was probably exerted by the remaining NK cells and other effector cells such as neutrophils and monocytes. The observed tumor ulceration seemed to be related to treatment efficacy. Only one ulcer was observed in the control mice and no ulcers were observed in the rituximab-treated mice, whereas the number of ulcers increased with increasing doses of ^177^Lu-lilotomab. Ulceration could therefore be due to the accelerated tumor necrosis caused by the therapy. The probable cause of the observed ulceration is the proximity of the subcutaneous tumor xenografts to the mouse skin.

We have shown in previous studies that ^177^Lu-lilotomab can synergize with rituximab in rituximab-sensitive cell lines. In the current study, we have taken the analysis one step further and shown that synergy can also be observed in rituximab-resistant cell lines and that rituximab resistance might be partially reversed by combining rituximab with ^177^Lu-lilotomab. Further studies using different rituximab-resistant cell lines and animal models with an intact immune system might be of interest to generalize our findings and gain deeper insight into the mechanisms of action behind the observed synergy.

The current results further support the rationale underlying the current clinical phase 1b trial (Archer-1; NCT03806179) of combination treatment of patients with relapsed or refractory follicular lymphoma and suggest that in the future ^177^Lu-lilotomab radioimmunotherapy could potentially be used for resensitization of relapsed or refractory NHL patients to CD20-targeting therapy.

## CONCLUSION

In this present work, we have demonstrated that radioimmunotherapy with ^177^Lu-lilotomab has the potential to reverse rituximab resistance through increased rituximab binding and ADCC activity in rituximab-resistant NHL models.

## DISCLOSURE

Betalutin (Nordic Nanovector) is currently being tested in a global phase 2b clinical trial for treatment of relapsed or refractory follicular lymphoma (NCT01796171), and the combination with rituximab is being tested in a phase 1 clinical trial (NCT03806179). The studies were funded by Nordic Nanovector and the Research Council of Norway under the Industrial PhD program, project 260203. Marion Malenge, Sebastian Patzke, Jostein Dahle, and Ada Repetto-Llamazares are employed by Nordic Nanovector. Ada Repetto-Llamazares is the author of a patent related to antigen upregulation by radioimmunotherapy. Ada Repetto-Llamazares, Trond Stokke, and Jostein Dahle own shares in Nordic Nanovector. Jostein Dahle is a member of the company’s leadership team. No other potential conflict of interest relevant to this article was reported.

**KEY POINTS**

**QUESTION:** Can ^177^Lu-lilotomab reverse rituximab resistance and improve the efficacy of rituximab therapy?
**PERTINENT FINDINGS:** ^177^Lu-lilotomab significantly increases rituximab binding and rituximab-mediated ADCC activity and, when used in combination with rituximab, has the potential to synergistically suppress tumor growth in an NHL mouse model.
**IMPLICATIONS FOR PATIENT CARE:** ^177^Lu-lilotomab could potentially be used for resensitization of relapsed or refractory NHL patients to CD20-targeting therapy.
