# Supplementary material for: 177Lu-Lilotomab Satetraxetan Has the Potential to Counteract Resistance to Rituximab in Non-Hodgkin Lymphoma
Source: J Nucl Med. 2020 Oct;61(10):1468–75. doi: 10.2967/jnumed.119.237230 (PMC7539655; doi:10.2967/jnumed.119.237230)
Supplement: Supplementary file 1 [file jnm237230SupplementaryData.pdf]

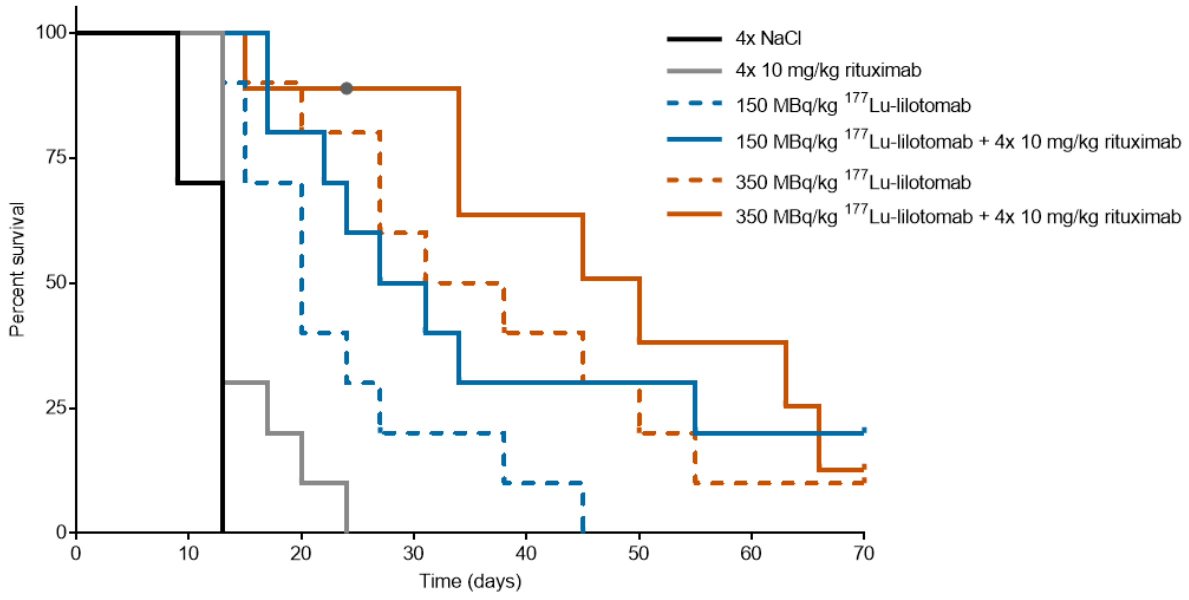

Supplemental Fig.1 Kaplan-Meier survival curves of Raji2R-xenografted mice treated with saline, rituximab, 150 MBq/kg and 350 MBq/kg ^{177}Lu-lilotomab monotherapy or combination with rituximab. N=10. Gray dots: censored animals.

Supplemental Table 1 Median survival time of mice treated with NaCl, rituximab, 150 and 350MBq/kg ^{177}Lu-lilotomab and combination therapies.

Treatment Group	Median survival ± SE (days)
4×NaCl	13±0
4×10mg/kg rituximab	13±3
150MBq/kg ^{177}Lu-lilotomab	20±3*,†
350MBq/kg ^{177}Lu-lilotomab	38±9*,†
150MBq/kg ^{177}Lu-lilotomab + rituximab	27±6*,†
350MBq/kg ^{177}Lu-lilotomab + rituximab	50±7*,†

*Significantly different from NaCl (p<0.001)

†Significantly different from 4x10 mg/kg rituximab (p<0.01)

Supplemental Table 2 Bliss synergy interaction values

	Interaction Value* (90% confidence interval)	p-value
150 MBq/kg ^{177}Lu-lilotomab + rituximab	0.7 (0.43-1.25)	0.54
350 MBq/kg ^{177}Lu-lilotomab + rituximab	1.58 (0.61-4.08)	0.43

*calculated using the hazards found through Cox Proportional Hazards model fitting to mouse survival.

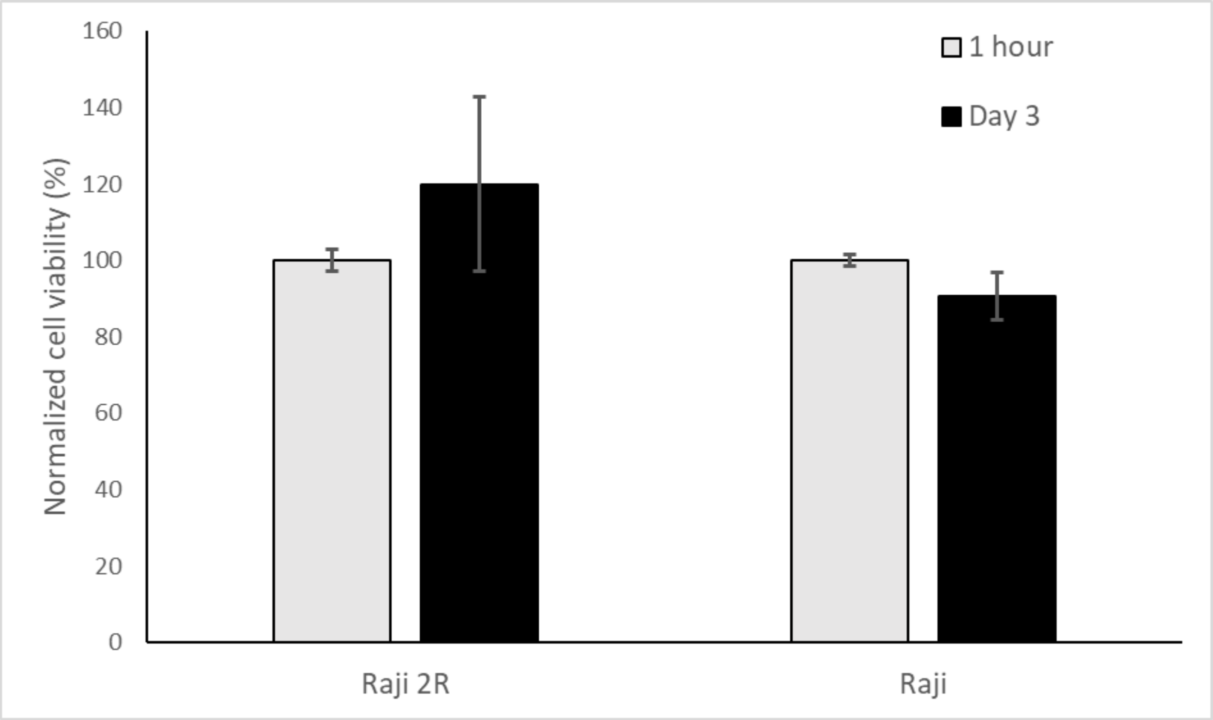

Supplemental Fig. 2 Effect of rituximab-treatment on viability in Raji and Raji2R cells. Mean ±SD, N=2

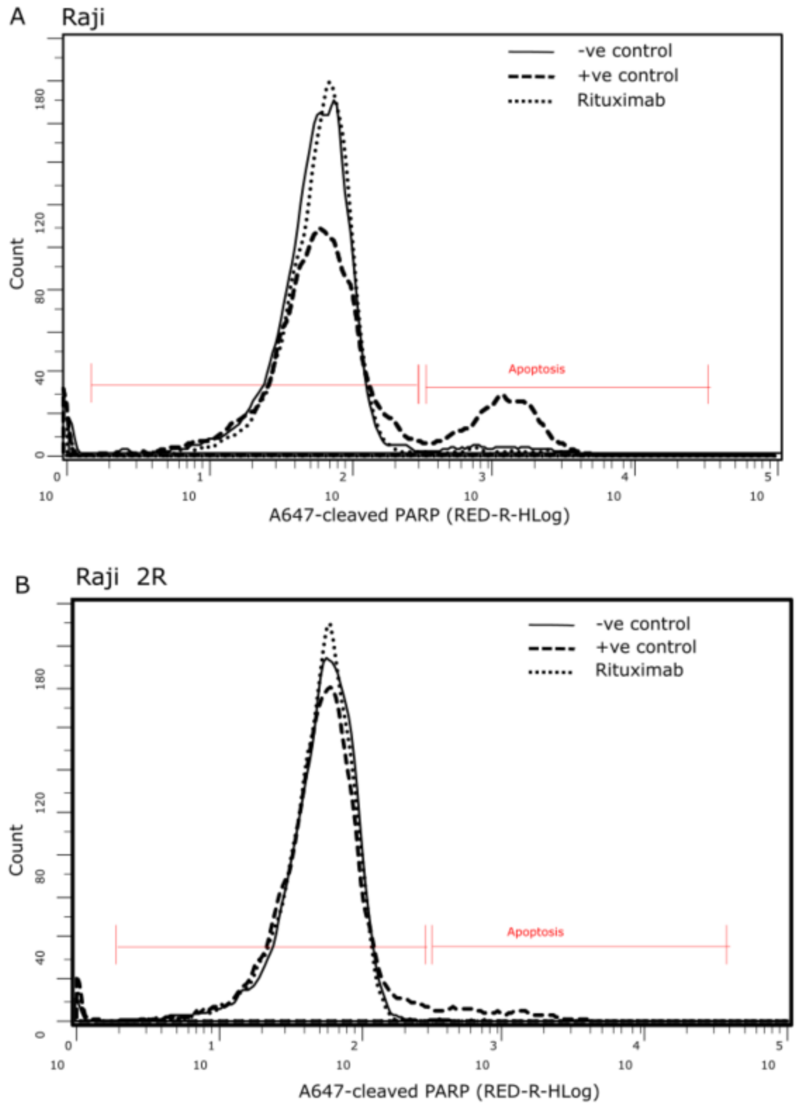

Supplemental Fig.3 Example histograms showing the change in induction of apoptosis after treatment of Raji and Raji 2R cells with PBS as a negative control, etoposide as a positive control denoted as -ve and +ve control respectively or 50µg/ml rituximab.
